# Reference materials for food authentication

**DOI:** 10.1007/s00216-025-05743-0

**Published:** 2025-01-29

**Authors:** Franz Ulberth, Robert Koeber

**Affiliations:** Joint Research Centre, European Commission, 2440 Geel, Belgium

**Keywords:** Food authenticity, Food fraud, Reference materials, Reference samples, Untargeted testing methods, Method validation

## Abstract

The global food industry faces significant challenges in ensuring the safety and authenticity of food products. Economic adulteration and counterfeiting of food are estimated to cost the industry US$30–40 billion annually. Analytical testing plays a vital role in detecting food fraud. For ensuring the metrological traceability and comparability of testing results, the use of reference materials (RMs) is crucial. The article describes the role of RMs in food authenticity testing, including their applications in method validation, calibration, quality control, and the definition of conventional measurement scales. It also reviews the availability of RMs that can be used in measurement procedures to authenticate food. Furthermore, the applications of RMs in targeted adulterant detection methods, for compositional parameters used to authenticate foods and food supplements, isotopic measurements, untargeted food authenticity testing methods, and detection and quantification of genetically modified organisms (GMOs), are explored. The document concludes by recommending the development of research grade test materials or representative test materials to harmonise untargeted testing methods and improve comparability of results across laboratories and over time.

## Introduction

Economic adulteration and counterfeiting of food products are a global problem with an estimated cost for industry of US$30 to US$40 billion a year [1]. The full extent of the problem is unknown because those who commit food fraud want to avoid detection and do not intend to cause physical harm. Still, such criminal negligence may endanger public health (cf. the melamine in milk incidence). Therefore, most fraudulent activities pass unnoticed and are not recorded. The complexity of modern food supply chains, which often span multiple countries and involve numerous intermediaries, makes them vulnerable to fraud. The longer and more complex the supply chain, the harder it is to trace and verify the authenticity of products. To address these vulnerabilities, stakeholders of the supply chain must enhance chain transparency, improve food traceability, implement robust control measures, and strengthen regulatory frameworks and enforcement mechanisms.

Legal definitions of what constitutes food fraud do not exist in most jurisdictions although the generic definition of food fraud as ‘intentional deception for economic gain using food’ is widely accepted [2]. The US FDA [3] as well as the European Commission [4] came up with more detailed working definitions, i.e. food fraud is a non-compliance concerning any suspected intentional action by businesses or individuals, for the purpose of deceiving purchasers and gaining undue advantage therefrom, in violation of the rules. The basic principle constituting food fraud is the intentional mismatch between food product claims and food product characteristics [5]. A product characteristic can be inherent in the product itself (e.g. composition); it can relate to the conditions of how the food was produced (e.g. organic, wild-caught), or the environment in which it was produced (e.g. geographical origin). Certain characteristics, such as the content of polyunsaturated fatty acids in fish oil, are directly amenable to measurements, whereas other characteristics (e.g. geographical origin) are not directly measurable. For the latter, appropriate marker substances, e.g. stable isotopes of light and heavy elements, are measured as proxies, and related to the characteristic of interest, i.e. geographical origin, by computational techniques.

Analytical strategies to authenticate food build either on (a) a fundamental difference between otherwise similar foods/ingredients, or (b) the empirical differences of the quantity of individual compounds, or (c) a broad range of identified and unidentified compounds contained in them. An example for (a) is 16-O-methylcafestol, which allows to discriminate Arabica from Robusta coffee, and the empirically known water-to-protein ratio of meat and fish species for detecting undeclared water addition is an example for (b). Quite often, a broad range of analytically determined compounds in combination with advanced data analysis is required for product classification (‘foodomics’). Whenever a classification is based on empirical differences, samples of the authentic products, often called reference samples, with demonstrated traceability to the product characteristic (geographical origin, husbandry system, production process, etc.) are a prerequisite for training an algorithm that discriminates them from similar, non-authentic products. A workshop organised by the National Institute of Standards and Technology (NIST) in 2019 highlighted the limited availability of test materials of known origin and growth conditions for many commodities as a bottleneck to the collection of data and development of data repositories for evaluation of food authenticity [6]. Likewise, the AOAC-Sponsored Workshop Series Related to the Global Understanding of Food Fraud (GUFF) identified an urgent need to develop reference materials (RMs) for food commodities that are prioritised for standardisation. Such materials are essential for global harmonisation of analytical methods to discern between deliberate fraud incidents and non-deliberate occurrence or contamination [7].

The purpose of this communication is to reflect on the metrological role of (certified) reference materials ((C)RMs) in the assessment of food authenticity, i.e. whether claims made on a food product are correct. Furthermore, the current availability of dedicated (C)RMs for use in food authentication is briefly reviewed and opportunities for future developments are identified.

## The role of reference materials in food authentication

Quite often, the terms ‘reference materials’ and ‘reference samples’ are used interchangeably in the food authenticity/fraud community, which may lead to misunderstandings, particularly of the metrological role of (C)RMs in authenticity testing. ISO Guide 30:2015 [8] defines a RM as ‘a material, sufficiently homogeneous and stable with respect to one or more specified properties, which has been established to be fit for its intended use in a measurement process’, and a CRM as ‘a RM characterized by a metrologically valid procedure for one or more specified properties, accompanied by an RM certificate that provides the value of the specified property, its associated uncertainty, and a statement of metrological traceability’. A note to the RM definition explains that properties can be quantitative or qualitative, e.g. identity of substances or species. Likewise, the concept of certified value may include a nominal property or a qualitative attribute such as identity or sequence; uncertainties for such attributes may be expressed as probabilities or levels of confidence. The International Vocabulary of Metrology (VIM) [9] provides similar definitions of RM and CRM but goes even beyond by requesting that the specifications of a RM should include its material traceability, indicating its origin and processing. Next to metrological traceability, the ‘traceability of nominal property values’ of CRMs should be given as well. ISO 33406:2024 specifically describes the terminology related to RM with qualitative property values and provides guidance on value assignment, assessment of homogeneity and stability, statement of metrological traceability, and measurement uncertainty [10]. Of particular relevance for food authenticity RMs is the concept of ‘provenance’, which requires documented information of the origin of the material, supported by additional evidence to confirm identity of the material, for value assignment [11].

Main applications of RMs are method validation (assessment of precision and bias of a measurement procedure), calibration, quality control, defining conventional measurement scales, and establishing metrological traceability [12]. All of the mentioned uses are relevant for food authenticity testing, albeit to a varying degree, depending on the type of testing. A fundamental difference (absence/presence of a marker compound) between otherwise similar food products lends itself for targeting the marker using an appropriate measurement procedure. If such a fundamental difference does not exist, one or more quantitative or qualitative markers in combination with data analytics is necessary for product discrimination/classification. Univariate evaluation of measured quantity values of markers is applicable if the classification is based on minimum or maximum threshold value(s) for the marker(s) or their arithmetic derivatives (ratios, differences, etc.). In this case, the analytical verification of authenticity is possible via a product characteristic that is directly measurable (i.e. targeted analysis). Otherwise, if the product characteristic cannot be inferred from a directly measurable marker, several measured quantity values of markers or information derived from electronic instrument records (‘features’), mostly spectra, have to be employed for the indirect assessment of product authenticity (i.e. untarget analysis).

The role of RMs in authenticity testing is summarised in Table 1. Depending on their function, RMs can be divided into RMs with metrologically traceable property values, which are used for validation/verification/quality control of measurement methods, and RMs with traceability of nominal property values such as authenticity (‘true to the name’), variety, geographical origin, or production system (material traceability). The latter are either used for determining the natural range of the marker compounds used for discriminating genuine from adulterated products, or to calibrate multivariate statistical models for classification. Evidently, RMs used for validation/verification/quality control of authenticity testing have to fulfil the homogeneity and stability requirement of the ISO Guide 30 definition [8]; practicability considerations can limit this requirement for RMs with traceable nominal properties. The key property of such RMs is their material and documentary traceability to the process, e.g. a specific production system in a given geographical area, which distinguishes them from otherwise similar products. A large number of them is necessary either for estimating the natural variation of a directly measurable product characteristic or for the indirect estimation of the characteristic through calibrated and validated statistical models.

**Table 1:** Reference material use in various measurement methods for food authentication. Profiling and fingerprinting are untargeted testing approaches, where ‘profiling’ means that individual marker substances are quantified and ‘fingerprinting’ means that spectral data are used without annotation of signals and quantitation of substances

RM with traceable material properties used for	Testing mode	Product characteristic	Example
Determination of the natural variation of marker substance(s)	Targeted	Authenticity (‘true to the name’)	16-O-methylcafestol marker for Arabica coffee
		Geographical origin	^87^Sr/^86^Sr isotope ratios for various commodities of plant origin
Determination of the natural variation of substances/features to calibrate the mathematical classification model	Profiling	Authenticity (‘true to the name’)	Triacylglycerol profile for purity estimation of cocoa butter
		Geographical origin	Element profile for various food products (often in combination with stable isotope ratio analysis)
		Production system (e.g. organic, wild-caught)	Fatty acid profile for organic eggs, wild-caught fish
	Fingerprinting	Authenticity (‘true to the name’), Geographical origin, Production system (e.g. organic, wild-caught)	NMR, MS, IR based metabolomics for various food products (wine, honey, spices, etc.)

Traditionally, national metrology institutes (NMIs), their designated institutes, or other accredited RM providers issue RMs with metrologically traceable property values. In order to ensure metrological traceability of the property values based on chemical and biological measurements, these institutions aliquot a larger amount of a candidate RM into units (ampoules, bottles, sachets, etc.), which are offered to the community over a long time. Next to the characterisation of the property value, this requires quite extensive homogeneity and stability studies to verify that the property value is within certain limits in each individual unit and sufficiently stable until the stated expiry date of the material. RMs that are used to characterise the natural compositional variation among individual items of a genuine product are often used only once for the determination of the characteristic of interest. The obtained measurement results, e.g. a molecular fingerprint, translate the physico-chemical properties of the material into electronic records. They are stored in a database together with meta-data describing material and documentary traceability (e.g. geographical origin, variety, production process) and form the basis for the authentication of unknown samples. Homogeneity and stability of these RMs are, therefore, of secondary importance, unless they are used in an interlaboratory comparison to validate an authenticity testing method. Nevertheless, appropriate procedures for sub-sampling from a larger quantity of material to arrive at a representative test portion have to be applied as well to assure data quality.

A food authenticity database is a repository of compositional data either in the form of quantity values or spectral information obtained from a representative number of authentic samples that are analysed with validated methods to define the natural variability of certain characteristics of a food. This natural variability forms the basis for computing the probability that an unknown sample belongs to the group of authentic samples. Collection of data and development of databases for evaluation of authenticity are a resource-intensive process. If done by commercial providers, it is understandable that they wish to recover expenses by charging fees for using their services. Quite often, the processes for creating the databases and the associated material traceability data are not publicly accessible, which obviously limits their transparency, and, consequently, their utility for official control purposes.

General guidance for building and curating food authenticity databases is available [13]. Obviously, the main aim is to obtain truly authentic samples, i.e. RMs, which are representative for the studied food product. Ideally, such RMs should be collected at the beginning of the supply chain, i.e. from primary producers (farms, fisheries, etc.), by impartial collectors to ensure that traceability and integrity of the RMs are maintained.

**Fig. 1:**
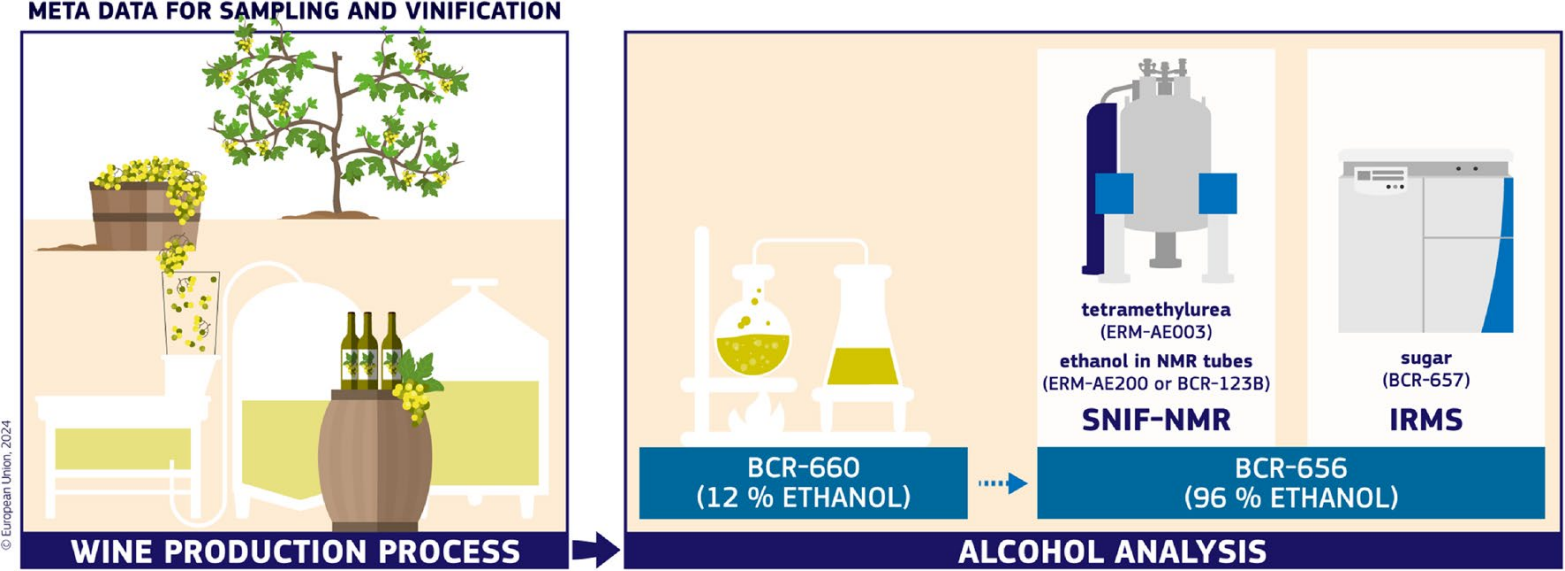
Use of CRMs for ensuring data quality in the EU Wine Database. ERM-AE003 (tetremethylurea) is an internal standard with a certified D/H amount-of-substance, BCR-660 (wine ethanol, 12% v/v) for control of the distillation step, and BCR-656 (wine ethanol, 94% m/m) for control of the SNIF-NMR and IRMS determination step. In addition, ERM-AE200 or BCR-123B (wine ethanol in NMR tubes to which TMU as internal standard and hexaflurobenzene as NMR lock substance) is used for performance verification of NMR spectrometers. BCR-657 (sugar) is used for traceability to the international Vienna-Pee Dee Belemnite (VPDB) scale

An exemplary model of a well-curated databank is the EU Wine Databank, which helps detect fraud in the wine sector [14]. It can be used as hard evidence to support the work of expert witnesses in court cases. It is a repository of wine ethanol and wine water isotopic data used to uncover undeclared sugaring of must and wine watering. EU legislation related to wine requires that every year officials collect grapes in vineyards in the wine-producing EU Member States, which are microvinified to produce reference wines with an unbroken material traceability chain. They are then analysed and the results submitted to the EU Wine Databank. Data repositories can only benefit users if the collected measurement data are comparable through space and time. Therefore, data providers (the competent authorities of the EU Member States) to the EU wine databank have to be ISO/IEC 17025 accredited, participate in dedicated proficiency testing rounds, use methods standardised by the International Organisation of Vine and Wine (OIV), and use CRMs to ensure traceability and comparability of the isotopic data submitted to the EU Wine Databank. The laboratories of the competent authorities use a suite of CRMs for establishing metrological traceability of isotopic measurements to the international isotopic measurement scales (Fig. 1).

This way of working is obviously quite resource intensive and it is, therefore, not frequently applied.

## Reference materials for targeted adulterant detection methods

These reference materials are used to calibrate, validate, and verify analytical methods for the identification of specific adulterants. Usually, they are prepared by mixing the adulterant and the authentic material in relevant proportions.

In the aftermath of the horse meat incidence, where undeclared horse meat was found in beef patties and ready-made meals, LGC Ltd. issued a series of meat RMs, among them horse, beef, pork, chicken, turkey, and meat mixtures (e.g. 1 and 5% beef in sheep meat) for the development and validation of meat speciation methods. Those RMs were made available on a one-off basis to address a topical problem and are no longer available. However, if needed in the future, production can be restarted at relatively short notice (LGC Ltd., personal communication).

Other examples are skim milk powders containing melamine at levels of 1, 400, 1000, and 5000 mg/kg, which are offered by the US Pharmacopeia. For the preparation of the powders, fluid skim milk was spray-dried in the presence of melamine to ensure commutability to industrially prepared powders. The National Institute of Metrology of China developed a similar set of melamine in milk powder CRMs (GBW10059 0.45 mg/kg, GBW10060 3.47 mg/kg, and GBW10061 16.5 mg/kg). BCR-599 (mixture of ewes’/goats’ curds containing either 0 or 1% (mass fraction) cows’ milk) was designed to verify an isoelectric focusing method to detect adulteration of ewes’ or goat’s cheese by cow’s milk.

The offer of such (C)RMs by RM producers is rather limited as the preparation of adulterant/authentic mixtures is in many cases straightforward, e.g. substitution of a part of olive oil by seed oils; therefore, they are usually prepared in-house and not purchased from a RM producer.

## Reference materials for compositional parameters used to authenticate foods and food supplements

Authentication of foods or food supplements often involves analysing the concentration ranges of specific constituents (biomarkers). Determining the fatty acid profile of named animal oils, including fish oil, and vegetable fats and oils, including olive oil, has a long tradition for checking the compliance of the fats and oils, e.g. with the specifications of the FAO/WHO Codex Alimentarius [15–18]. To support measurements related to food quality and, in particular, for implementing provisions for mandatory nutrition labelling, several CRMs are available for validating/verifying and quality assuring the underlying measurement methods for fatty acid profiling (Table 2). Fatty acid profiles are less suitable for detecting fat/oil adulteration if the fatty composition of the genuine product and the adulterant is similar. For example, the presence of tallow and lard in milkfat and illipe and shea in cocoa butter is difficult to detect by fatty acid analysis. Triglyceride (TG) profiling forms a better-suited alternative and to support this approach the European Commission’s Joint Research Centre (EC-JRC) developed BCR-519 (Anhydrous butter fat) and IRMM-801 (cocoa butter). The certified properties of those CRMs are TG profiles (g TG/100 g total TG) determined by gas–liquid chromatography with flame ionisation detection. Both are part of a ‘tool kit’ consisting of the CRMs to calibrate a standardised measurement method, ISO 17678:2019 | IDF 202:2019 for milk fat [19] and ISO 23275–1:2006 for cocoa butter [20], which also describe an algorithm for purity assessment of the fats, i.e. detecting whether foreign fats are present. For the development of the algorithms, the standardised methods, which prescribe the use of BCR-519 and IRMM-801 for instrument calibration, were used to determine the TG profiles of a large number of authentic milkfats and cocoa butters [21, 22].

**Table 2:** (C)RMs to support fats and oils analysis (*EU-JRC*, European Commission-Joint Research Centre; *NIST*, National Institute of Standards and Technology)

Producer	(C)RM	Name	Certified properties
EU-JRC	BCR-162R	Soya-maize oil blend	Fatty acid methyl ester (FAME)/100 g total FAMEs
	BCR-164^a^	Milkfat	FAME/100 g total FAMEs, butyric acid (mass fraction)
	BCR-519^b^	Milkfat	Mass fraction of triglycerides (TG)/100 g total TG
	BCR-633	Anhydrous butter fat	Sitosterol, stigmasterol, n-heptanoic acid triglyceride, β-apo-8′-carotenic acid ethyl ester
	IRMM-801^c^	Cocoa butter	Mass fraction of TG/100 g total TG
NIST	RM 8037	Krill Oil	Mass fraction of fatty acids (as free fatty acids)
	RM 8182	Fatty acid methyl esters in 2,2,4-trimethylpentane	26 Fatty acid methyl esters
	RM 8183	Omega-3 and omega-6 fatty acids in botanical oils 8183–1 Borage, 8183–2 Evening Primrose, 8183–3 Flax, 8183–4 Perilla	Mass fraction of fatty acids (as triglycerides)
	SRM 3275	Omega-3 and omega-6 fatty acids in fish oil 3275–1, concentrate high in docosahexaenoic acid (DHA); 3275–2, anchovy oil high in DHA and eicosapentaenoic acid (EPA), 3275–3, concentrate containing 60% long-chain omega-3 fatty acids	Mass fraction of fatty acids (as FAMEs)

^a^Sold out; replacement material ERM-BD164 in preparation

^b^Sold out; replaced by new batch ERM-BD519

^c^Sold out; replacement material ERM-BD801 in preparation

BCR-633 (anhydrous butter fat — tracers) is certified for β-sitosterol (530 mg/kg) and stigmasterol (147 mg/kg) and can be used to verify analytical methods for detecting an undeclared addition of vegetable fats/oils to milk/butter fat. The content of butyric acid is a traditionally used method to determine the milk fat content in fat mixtures. BCR-164 (anhydrous milk fat), currently sold out and undergoing re-production, can be used to either calibrate or validate the required detection method.

The safety and quality of botanical products such as herbal medicines and food supplements depend largely on the authenticity of the raw materials used for their preparation. The adulteration of botanical products is a growing problem. A bibliographic study found 27% of 2386 commercial herbal products, sold in 37 countries spread over six continents, to be adulterated [23]. Macroscopic (plant morphology/anatomy), microscopic (plant histology), and sensory evaluation alongside chemical and DNA-based assays are the tools for authentication of botanicals [24]. However, botanicals are quite often traded as processed products (powders or extracts), where the characteristic macroscopic and microscopic features are no longer discernible, making chemical or molecular biology techniques the only option for authentication. The phytochemical makeup of a botanical, in particular marker compounds, facilitates the assessment of its (chemo)taxonomic identity. Pharmacopoeial monographs or guidance documents provided by herbal product associations contain the required qualitative and/or quantitative chemical information and specify assays for purity and authenticity. (C)RMs in the form of pure biomarker substances, and extracts and plant parts with documented material traceability are crucial for assessing botanical authenticity as they allow a direct comparison of an authentic material to a test sample [24–26] (Table 3). Obviously, the possible variation of the phytochemical composition of the test sample due to pedo-climatic, agronomic, etc., factors has to be accounted for in the assessment.

**Table 3:** NIST CRMs and RMs to support authentication of botanicals. Extracts and oral dosage forms of the listed botanicals with values assigned for phytochemicals and elements are also available

(C)RM	Name	(Certified) Properties (mass fractions and genetic information)
RM 8650	Ground Kudzu (*Pueraria montana* var. *lobata*) Rhizome	Isoflavones (puerarin, daidzin, daidzein) and As, Cd, Pb Species identity (Sanger sequencing of chloroplast *trnL-trnF* and nuclear ribosomal ITS2 region), DNA sequences as FASTA-formatted files
SRM 3246	*Ginkgo biloba* (Leaves)	Flavonoids (quercetin, kaempferol, isorhamnetin, total aglycones), terpene lactones (ginkgolides A, B, C, J, bilobalide, total terpene lactones), toxic elements (Cd, Pb, Hg) Species identity (Sanger sequencing of chloroplast *psbA-trnH* and *trnL* region), DNA sequences as FASTA-formatted files
SRM 3250	Saw Palmetto (*Serenoa repens*) Fruit	Phytosterols (campesterol, sitosterol, stigmasterol) Species identity (Sanger sequencing of chloroplast *psbA-trnH* and *trnL-trnF* region), DNA sequences as FASTA-formatted files
SRM 3254	Green Tea (*Camellia sinensis*) Leaves	Catechins and xanthines ((–)-epicatechin, (–)-epicatechin gallate, (–)-epigallocatechin, (–)-epigallocatechin gallate, (–)-gallocatechin gallate, caffeine, theobromine, ( +)-catechin, (–)-gallocatechin, gallic acid, L-theanine), elements (As, Cd, Hg, Pb, Al, Co, Fe, Mn, Zn) Species identity (Sanger sequencing of chloroplast *psbA-trnH* and nuclear ribosomal ITS region), DNA sequences FASTA-formatted files
SRM 3262	St. John’s Wort (*Hypericum perforatum* L.) Aerial Parts	Chlorogenic acid, flavonoids, naphthodianthrones (chlorgenic acid, rutin, hyperoside, quercetin, hypericin, pseudohypericin), elements (As, Cd, Hg, Pb) Species identity (Sanger sequencing of chloroplast trnL-*trnF* and *rbcL* and nuclear ribosomal ITS regions), DNA sequences as FASTA-formatted files
SRM 3281	Cranberry (Fruit)	Quinic acid, anthocyanidins (cyanidin, delphinidin, peonidin, proximates (solids, ash, protein, carbohydrates, total sugars, fructose, glucose), calories and elements (Ca, Cu, Fe, Mg, Mn, P, K, Na, Zn, Al, Cl)
SRM 3287	Blueberry (Fruit)	Quinic adic, free water-soluble vitamins (thiamine, niacin, pantothenic acid, pyridoxine), elements (Ca, Cu, Fe, Mg, Mn, P, K, Zn, Na) organic acids (galcturonic, glycolic, isocitric, oxalic, shikimic), phosphate, sulfate, anthocyanidins (cyaniding, delphinidin, malvidin, petunidin, peonidin), proximates (solids, ash, fat, protein, carbohydrate, total sugars, fructose, glucose, dietary fibre), calories, amino acids (alanine, arginine, aspartic acid, cysteine, glutamic acid, glycin, isoleucine, leucine, lysine, phenylalanine, proline, serine, threonine, tyrosine, valine)
SRM 3384	Ground Asian Ginseng (*Panax ginseng* C.A. Meyer) Rhizome	Ginsenosides (Rb1, Rb2, Rc, Rd, Re, Rf, Rg1), elements (As, Pb, Cd) Species identity (Sanger sequencing of chloroplast DNA *trnL-trnF* and nuclear ribosomal ITS2 region) and DNA sequences as FASTA files

Next to phytochemistry, DNA-based techniques, such as barcoding, melting analysis, and metabarcoding by Next Generation Sequencing, are attractive techniques for determining the identity of botanicals. However, the quality of the available DNA and the different amplification rates of DNA origination from contaminating species can limit the applicability of DNA assays [23].

US regulations [27] require that dietary supplements be evaluated for identity, purity, and strength as well as for contaminants (toxic elements, bacteria, persistent organic pollutants, and other toxins) and adulterants (either unintentional or economically motivated), placing increased emphasis on the importance of the development of dietary supplement CRMs. As a result, the NIST started the development of several suites of Standard Reference Materials (SRM®)^1^ to help dietary supplement manufacturers comply with the current Good Manufacturing Practice rules of the US Food and Drug Administration [28]. Depending on the product and the inherent analytical challenge, a suite may consist of a raw botanical material, an extract, and a finished product for oral dosage. The identities of most of the botanical (C)RMs listed in Table 3 were confirmed by a trained botanist and DNA sequencing as well as determination of the phytochemicals characteristic for the botanical material. They can be used to validate/verify test methods described in compendial monographs for assessing quality and authenticity of the respective botanicals.

Botanical RMs and related phytochemical RMs are offered by the US Pharmacopoeia®, American Herbal Pharmacopoeia®, Extrasynthese/BotaniCERT®, and ChromaDex as well. Botanical gardens and natural history museums, e.g. the Botanic Garden and Botanical Museum Berlin, Germany, or the Royal Botanic Gardens, Kew, UK, are other notable providers of DNA samples from underlying voucher specimens for verifying DNA assays for the identification of botanicals.

## Reference materials for isotopic measurements

The analysis of the stable isotope ratios of bio-(H, C, N, O, S) as well as radiogenic element (Pb, Sr) is a powerful tool for detecting food fraud and safeguarding consumer trust. The isotopic composition of food reflects its production environment (climate, altitude, latitude, geology), agronomical practices (e.g. organic vs. synthetic fertilisers, grain-based vs. grass-based feeding), and mode of carbon fixation (C_3_ vs. C_4_ vs. CAM plants). It helps in the verification of the geographical origin of food products [29], discrimination of organically from conventionally produced food [30], and detection of wine chaptalisation [31] or addition of sugar products to honey [32]. Primary isotopic RMs anchor and define the scales on which isotopic ratios are reported as deviations (*δ* values) from the anchor point. Types of primary isotopic RMs and their producers are summarised in [33]. Because their availability is limited and many are inorganic compounds, research agencies and NMIs developed secondary RMs based on foodstuffs to minimise matrix effects (Table 4) [34–37]. To obtain consistent and comparable results, stable isotope ratio analysis should obey the Identical Treatment (IT) principle of subjecting samples and RMs to the same chemical and physical processing [38]. In case that the available secondary RMs do not match the matrix of test samples, in-house standards chemically similar to the test samples can be prepared for QA/QC purposes; guidance for the preparation of such in-house RMs is available [39].

**Table 4:** Secondary reference materials for isotope-ratio analysis of foods (*USGS*, U.S. Geological Survey; *CAAS*, Chinese Academy of Agricultural Sciences; *NRC*, National Research Council Canada; *TÜBİTAK UME*, TÜBİTAK National Metrology Institute)

Producer	CRM	Name	Consensus *δ* values*
USGS	USGS82	Honey from tropical Vietnam	δ^2^H_VSMOW‑SLAP_, δ^13^C_VPDB‑LSVEC_, δ^18^O_VSMOW‑SLAP_
	USGS83	Honey from prairie in Canada	δ^2^H_VSMOW‑SLAP_, δ^13^C_VPDB‑LSVEC_, δ^18^O_VSMOW‑SLAP_
	USGS84	Olive oil from Sicily, Italy	δ^2^H_VSMOW‑SLAP_, δ^13^C_VPDB‑LSVEC_, δ^18^O_VSMOW‑SLAP_
	USGS85	Olive oil from coastal desert, Peru	δ^2^H_VSMOW‑SLAP_, δ^13^C_VPDB‑LSVEC_, δ^18^O_VSMOW‑SLAP_
	USGS86	Peanut oil from tropical Vietnam	δ^2^H_VSMOW‑SLAP_, δ^13^C_VPDB‑LSVEC_, δ^18^O_VSMOW‑SLAP_
	USGS87	Corn oil from the USA	δ^2^H_VSMOW‑SLAP_, δ^13^C_VPDB‑LSVEC_, δ^18^O_VSMOW‑SLAP_
	USGS88	Marine collagen from wild-caught fish	δ^2^H_VSMOW‑SLAP_, δ^13^C_VPDB‑LSVEC_, δ^15^N_AIR_, δ^18^O_VSMOW‑SLAP_, δ^34^S_VCDT_
	USGS89	Porcine collagen	δ^2^H_VSMOW‑SLAP_, δ^13^C_VPDB‑LSVEC_, δ^15^N_AIR_, δ^18^O_VSMOW‑SLAP_, δ^34^S_VCDT_
	USGS90	Millet flour from Tuscany, Italy	δ^2^H_VSMOW‑SLAP_, δ^13^C_VPDB‑LSVEC_, δ^15^N_AIR_, δ^18^O_VSMOW‑SLAP_, δ^34^S_VCDT_
	USGS91	Rice flour from tropical Vietnam	δ^2^H_VSMOW‑SLAP_, δ^13^C_VPDB‑LSVEC_, δ^15^N_AIR_, δ^18^O_VSMOW‑SLAP_, δ^34^S_VCDT_
CAAS	CAAS‐1801 CAAS‐1802	Defatted beef from China	δ^13^C_VPDB_, δ^15^_AIR_ δ^13^C_VPDB_, δ^15^_AIR_
NRC	VANA-1 VANA-2	Vanillin	δ^13^C_VPDB‑LSVEC_
	BEET-1	Beet sugar	δ^13^C_VPDB‑LSVEC_
TÜBITAK UME	UME 1312	Honey (unadulterated)	δ^13^C_VPDB_
	UME 1313	Honey (adulterated)	δ^13^C_VPDB_

^*^Subscripts denote the primary standards for anchoring the isotope delta scales reported in the certificates. *VSMOW*, Vienna Standard Mean Ocean Water; *SLAP*, Standard Light Antarctic Precipitation; *VPDB*, Vienna Pee Dee Belemnite; *LSVEC*, Li Svec; *VCDT*, Vienna Cañon Diablo Troilite

For product authentication, isotopic delta values of test samples are compared by statistical methods to values of reference samples with documented material traceability. Reference databanks of isotopic data for a number of food commodities exist but many are either curated and operated by commercial service providers, or their access is limited to authorised users. The latter is the case for the EU Wine Databank and for databanks created by producer associations, e.g. the Grana Padano Cheese Protection Consortium for their PDO cheese [40]. The FoodAuthenticity Network maintains a searchable list of isotopic databanks of food commodities, which can be used as reference [41].

## Reference materials for untargeted food authenticity testing methods

Untargeted metabolomics using different technology platforms has rapidly gained prominence in food authenticity testing [42]. To illustrate the popularity of untargeted testing, the Scopus® database was searched in March 2024 with the search string ‘food AND (fraud OR adulterat* OR authent*)’. This produced 7176 hits for 2018–2023 and refining them with the term ‘metabol*’ gave a reduced set of 2273 documents. This highlights the popularity of the approach as around one-third of the articles used metabolite features to decide whether the tested food conforms to its product specification.

Untargeted food authentication methods compare two or more datasets of metabolite features to find differences between them that discriminate authentic and non-authentic products. To achieve this, it is essential to separate variability between datasets into material differences and differences resulting from experimental variation and artefacts. Thus, the ability to identify and control intra- and inter-laboratory variability is a critical aspect of untargeted metabolomics. Initiatives such as the Metabolomics Standards Initiative (MSI) [43] and the Metabolomics Quality Assurance and Quality Control Consortium [44] have progressed the development of reporting standards, QA principles, and QC materials for producing high-quality data and fostering confidence in the obtained results. One of the standard practices in a metabolomics experiment is to combine aliquots from all experimental samples to form a QC pool, which is then analysed at regular intervals together with the experimental samples. This helps detect drift or irregularities in instrument performance over time but this approach is less useful for comparing and integrating datasets produced in different study centres. Mixtures of standard substances and the use of their isotopologues as internal standards are one way of ensuring data quality across laboratories and analytical instrumentation. Nonetheless, matrix-matched and value-assigned RMs are the preferred option as they are subjected to the same experimental variation as the study samples (IT principle). To eliminate the influence of experimental variation in untargeted metabolomics to the extent possible, workflows include a data pre-processing step, including signal intensity drift correction, before chemometric data analysis. By comparing the intensity of the metabolite features in the samples to the QC materials, ideally RMs, the data can be normalised to account for these variations. Signal drift correction is especially important for chromatography-linked mass spectrometry systems to adjust changes in metabolite feature response over time due to fluctuating instrument sensitivity. The ratio-based method, i.e. by scaling absolute feature values of study samples relative to those of concurrently processed RMs, was found to be more effective than other batch correction methods [45]. Transfer of calibration models among infrared spectrometers also profits from correction procedures that use common reference materials [46]. In contrast, NMR-based untargeted metabolomics is more robust, since instrument fluctuations and signal drift issues are negligible. With a given set of experimental conditions, the obtained spectra are highly reproducible across multiple instruments and laboratories and normalisation with matrix QC materials is generally not required [47]. Thus, the technique has been used to collect spectra and metadata of food commodities in databanks, which can be interrogated and used for predictive class modelling. An example of such an application is the FoodScreener™, a fee-for-service NMR solution for authenticity testing of wine, honey, fruit juice, and olive oil [48].

SRM® 1950 Metabolites in Human Plasma, released in 2011, was the first CRM developed specifically for metabolomics research. Internationally accessible QA/QC RMs for untargeted food authenticity testing are not widely available at the moment. Seafood is quite often misdescribed and tissue RMs for economically important species are needed to verify DNA-based methods for taxonomic identity testing. Only few CRMs such as wild-caught (NIST RM 8256) and aqua-cultured Coho salmon (NIST RM 8257), wild-caught shrimp (NIST RM 8258) and aqua-cultured shrimp (NIST RM 8259), and Atlantic halibut, pollack, and saithe (JRC EURM-020, JRC EURM-021, JRC EURM-022) are currently available (Table 5). FISH-FIT (https://www.fish-fit.org/about) offers authentic tissues of important European seafood species, including fish such as gadoids and tunas, but also molluscs and crustaceans. NIST RM 8256 and RM 8257 were value assigned for fatty acids and total fat content; genetic information is provided as well [49]. Next to that, their usefulness as matrix-matched QC materials in untargeted -omics applications to evaluate instrument precision, correct data due to instrument variation, and harmonise studies across laboratories was demonstrated. Multiomics analyses demonstrated that the protein, lipid, and metabolite fingerprints allowed discrimination between wild-caught and aqua-cultured salmon and shrimp. Besides this, the study also showed that freeze-drying the seafood material altered to some extent the metabolite composition compared to the fresh-frozen starting material [50].

**Table 5:** (C)RMs to support authentication of fish and seafood

EU-JRC	EURM-020	Atlantic halibut (*Hippoglossus hippoglossus*)	Species identity; consensus value of two barcodes (mitochondrial cytochrome b and mitochondrial cytochrome c oxidase subunit I gene) obtained by bidirectional Sanger sequencing
	EURM-021	Pollack (*Pollachius pollachius*)	Species identity; consensus value of two barcodes (mitochondrial cytochrome b and mitochondrial cytochrome c oxidase subunit I gene) obtained by bidirectional Sanger sequencing
	EURM-022	Saithe (*Pollachius virens*)	Species identity; consensus value of two barcodes (mitochondrial cytochrome b and mitochondrial cytochrome c oxidase subunit I gene) obtained by bidirectional Sanger sequencing
NIST	RM 8256	Wild-caught Coho Salmon (*Oncorhynchus kisutch*)	Fatty acids (myristic, palmitic, palmitoleic, stearic, oleic, vaccenic, linoleic, alpha-linoleic, stearidonic, eicosenoic, eicosodienoic, eicosapentaenoic, erucic, docosapentaenoic, docosahexaenoic, nervonic), monounsaturated fat, polyunsaturated fat, saturated fat, total fat, crude fat, total protein Species identity (Next Generation Sequencing of cytochrome c oxidase subunit 1 mitochondrial gene), phylogenetic analysis by amplifying the mitochondrial DNA (mtDNA) cytochrome c oxidase subunit III/ND3 (COIII/ND3) region for sequencing
	RM 8257	Aquacultured Coho Salmon (*Oncorhynchus kisutch*)	Fatty acids (myristic, pentadecanoic, palmitic, palmitoleic, margaric, heptadecenoic, stearic, oleic, vaccenic, linoleic, alpha-linolenic, gamma-linolenic, stearidonic, arachidic, eicosenoic, eicosodienoic, homo-gamma-linolenic, arachidonic, eicosapentaenoic, erucic, docosapentaenoic (n-3), docosapentaenoic (n-6), docosahexaenoic), monounsaturated fat, polyunsaturated fat, saturated fat, total fat, crude fat, total protein Species identity (Next Generation Sequencing of cytochrome c oxidase subunit 1 mitochondrial gene), phylogenetic analysis by amplifying the mitochondrial DNA (mtDNA) cytochrome c oxidase subunit III/ND3 (COIII/ND3) region for sequencing
	RM 8258	Wild-caught Shrimp	Fatty acids (palmitic, palmitoleic, stearic, oleic, arachidonic, eicosapentaenoic, docosahexaenoic), monounsaturated fat, polyunsaturated fat, saturated fat, total fat, crude fat, total protein Species identity (Next Generation Sequencing of cytochrome c oxidase subunit 1 mitochondrial gene), phylogenetic analysis by amplifying the mitochondrial mtDNA 16S region for sequencing
	RM 8259	Aquacultured Shrimp	Fatty acids (palmitic, stearic, oleic, linoleic, arachidonic, eicosapentaenoic, docosahexaenoic), monounsaturated fat, polyunsaturated fat, saturated fat, total fat, crude fat, total protein Species identity (Next Generation Sequencing of cytochrome c oxidase subunit 1 mitochondrial gene), phylogenetic analysis by amplifying the mitochondrial mtDNA 16S region for sequencing

Developing and producing matrix-matched (C)RMs are a resource-intensive endeavour and the time needed until the material is finally released can be considerable. For rapidly developing research areas, like the -omics, the timely provision of a common test material, termed Research Grade Test Material (RGTM) by NIST or Representative Test Materials by EC-JRC, facilitates measurement system suitability testing, intra- and interlaboratory method development, and between-method harmonisation. Such materials are particularly helpful to support measurement communities in developing and/or harmonising new methods [51]. Furthermore, if method developers provide feedback on the use of those materials and the obtained measurement results, valuable insights can be gained for the development of CRMs to address the needs of the community.

## Reference materials for the detection and quantification of genetically modified organisms (GMOs)

The labelling of GMOs, also termed bioengineered (BE) food in certain jurisdictions, is a contentious issue, with differing views on the safety and ethical implications of GMOs. In many countries, including the USA and those in the European Union, regulations require that food products containing GMO ingredients be labelled as such. Failing to disclose the presence of GMOs in food products, when required by law, can be seen as deceptive and fraudulent as it deprives consumers of the opportunity to make informed purchasing decisions based on their personal preferences and beliefs.

(C)RMs are essential for laboratories and institutions conducting GMO testing to ensure the quality and reliability of the testing results. They are either offered as matrix RMs in powder form, certified for mass fractions, or as genomic DNA (C)RMs and plasmid DNA (C)RMs, certified for copy number ratios. Several organisations such as the Joint Research Centre of the European Commission (https://crm.jrc.ec.europa.eu/), the American Oil Chemist’s Society (https://www.aocs.org/crm), the National Institute of Metrology, China (https://en.nim.ac.cn/), and NipponGene (https://nippongene.com/), play a crucial role in producing and distributing (C)RMs for the accurate and reliable detection of GMOs in food and feed. An overview of available GMO (C)RMs, their characteristics, strategies for GMO (C)RM development, and the institutions providing them is provided by Wu et al. [52]. Updated lists of available (C)RMS can be found on the web pages of the mentioned RM producers.

## Outlook

(C)RMs play a crucial role in ensuring the metrological traceability and, consequentially, the comparability of testing results. Independent of the laboratory setting, they are used in method validation/verification, calibration, and quality control, and in the definition of conventional measurement scales. By analogy to the latter function, RMs for untargeted food authenticity testing also define a ‘scale’, i.e. the natural variation of one or more characteristics of authentic products, to grade the similarity of a test item to truly authentic products. Sourcing of RM with traceable material properties for creating a food authenticity database is a resource-intensive process. It needs careful planning, engagement of food chain stakeholders, and appropriate funding. Producer organisations, service providers, and the competent authorities are the main actors responsible for providing traceable materials. Depending on the scope of an authenticity programme, a collaborative approach to laboratory testing can be an option to lessen the burden for a single laboratory. In such a scenario, reproducibility across multiple laboratories participating in the programme is a critical challenge. RMs that fulfil the requirements of ISO Guide 30 are thus critical QC tools to be able to assure reproducibility of untargeted food authenticity testing data. The community would certainly profit from the availability of Research Grade Test Materials or Representative Test Materials to harmonise untargeted testing methods and improve comparability of results across laboratories and over time. Honey, olive oil, and wine would be good candidates for making such materials available as they are frequently targeted by fraudsters and untargeted testing methods have a high potential to detect various types of adulterations in those commodities.

## Footnotes

1 NIST Standard Reference Material® (SRM) — A CRM issued by NIST that also meets additional NIST-specific certification criteria and is issued with a certificate or certificate of analysis that reports the results of its characterisations and provides information regarding the appropriate use(s) of the material.
